# Core–shell coaxially structured triboelectric nanogenerator for energy harvesting and motion sensing†

**DOI:** 10.1039/c7ra12739a

**Published:** 2018-01-15

**Authors:** Zhumei Tian, Jian He, Xi Chen, Tao Wen, Cong Zhai, Zengxing Zhang, Jundong Cho, Xiujian Chou, Chenyang Xue

**Affiliations:** Science and Technology on Electronic Test and Measurement Laboratory, North University of China Taiyuan 030051 China xuechenyang@nuc.edu.cn; Department of Electronics, Xinzhou Teachers University Xinzhou 034000 China; Department of Micro and Nano Systems Technology, University College of Southeast Norway Horten 3184 Norway; Department of Electrical and Electronic Engineering, Sungkyunkwan University Suwon 16419 South Korea

## Abstract

Converting sustainable human motion energy into electric energy has become an urgent task for the advancement of next-generation wearable and portable electronics. Herein, a core–shell coaxially structured triboelectric nanogenerator (CSTN) was fabricated by inserting an inner hollow circular tube into an outer hollow circular tube, and a gasbag is constructed within the space between the inner and outer tubes. Both Ni-coated polyester conductive textile and the conductive silicone rubber were used as effective electrode materials. The CSTN has excellent properties, including flexibility, light weight, sustainability and biological compatibility due to its unique structural design and materials selection. The CSTN can convert various forms of human motion energy, such as pressing, bending and twisting motion, into electric energy. A high short-circuit current of 11 μA and an open-circuit voltage of 380 V can be obtained from a CSTN with a length of 6 cm, corresponding to a high peak power of 1.638 mW at a load resistance of about 10 MΩ. When six such CSTNs are connected in parallel and placed under shoes, the electric energy output by normal walking can light up 60 LEDs connected serially and power up a competition-timer. The device can also sense different bending angles or twisting angles according to its signal outputs under different deformation angles. This study indicates the promising application prospects of the CSTN for next-generation devices, including self-powered illuminating devices, portable electronics, body motion sensing and health monitoring.

## Abstract 2

A core–shell coaxially structured triboelectric nanogenerator (CSTN) is fabricated by inserting the inner hollow circular tube into the outer hollow circular tube, which can be used for energy harvesting and motion sensing.

## Graphical abstract

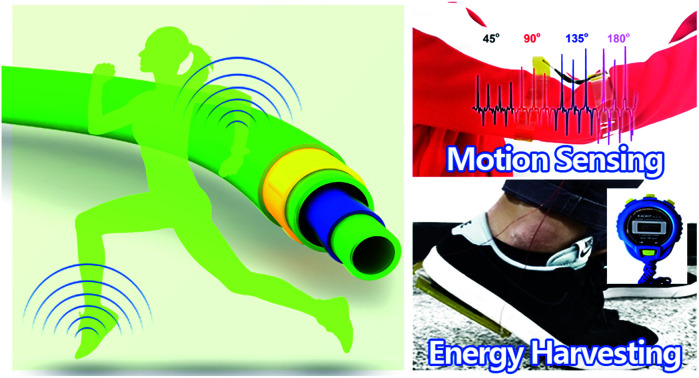

## 1. Introduction

In recent years, many new types of wearable electronics, such as intelligent glasses, smart bracelets, health monitoring instruments, exercise state monitoring equipment and implantable electronic chips, are being widely used in our daily life. With an increase in market demand for wearable and portable electronics, it is still a great challenge to provide flexible, sustainable and eco-friendly power sources for the above mentioned devices.

Many researchers have been working on energy harvesting devices using the thermoelectric effect,^1^ photoelectric effect,^2,3^ electromagnetic interaction,^4^ piezoelectric effect^5–7^ and triboelectric effect.^8,9^ Among the various energy harvesting methods mentioned above, the triboelectric nanogenerator (TENG) has received widespread attention for its ability to harvest energy from the surrounding environment, for example, water-wave energy,^10,11^ wind energy,^12^ rain energy,^13,14^ vibration energy^15–17^ and human motion energy.^18–23^ In addition, it also has the unique advantages of small size, high output performance and low cost, and has been used in healthcare,^24,25^ sensor application,^26–28^ as an energy source^29,30^ and so forth.

Motion energy can be harvested from almost any form of human motion, including walking, running, flapping, and touching. Some studies have been conducted to convert sustainable human motion energy into electric energy through triboelectric nanogenerator (TENG), which is considered as one of the most effective ways to solve the power supply problem for wearable electronics. Seung *et al.* invented a nanopatterned textile-based wearable triboelectric nanogenerator with a high power-generating performance.^31^ Ko *et al.* reported a multi-stacked PDMS-based triboelectric generator for efficient energy harvesting,^32^ while Tian *et al.* introduced a double-layer-stacked triboelectric textile for harvesting human motion energy.^33^ However, most of these devices are presented as planar structures, which make it difficult for them to be integrated with clothing and adapt to human motion in diverse directions.

In contrast, tube-based and fiber-based triboelectric nanogenerators have better flexibility, and are also expected to be used in implantable devices, health monitoring, biological medical observation and sensing systems in future. Yu *et al.* demonstrated a coaxial triboelectric nanogenerator fiber for energy harvesting and sensing under deformation.^34^ Wang *et al.* developed tube-like TENGs to drive wearable electronic devices.^30^ Sim *et al.* studied a stretchable triboelectric fiber for self-powered kinematic sensing.^18^ Zhong *et al.* introduced a fiber-based generator for wearable electronics and mobile medication.^22^

In this study, we demonstrate a triboelectric nanogenerator with a unique core–shell coaxial structure (CSTN) for energy harvesting and motion sensing with the advantages of flexibility, light weight, sustainability and biological compatibility. The designed CSTN consists of a hollow inner tube and a hollow outer tube, which are considered as the core and shell of the CSTN, respectively. The entire structure is encapsulated by silicone rubber that protects the device from ambient contamination, and then air is injected into the space between the inner tube and the outer tube to form a gasbag, which can effectively improve the friction power generation performance. Moreover, it can effectively convert various forms of human motion energy (pressing motion, twisting motion and bending motion) into electric energy. It can also sense different bending angles or twisting angles according to its signal outputs under different deformation angles. Therefore, it is not only able to work as an energy harvesting unit, but also acts as a sensor unit to detect bending angles or twisting angles, which indicates its broad application prospects in self-powered illuminating devices, portable electronics, motion sensing, health monitoring and so on.

## 2. Experimental

### 2.1 Material preparation

The silicone rubber mixture was prepared by uniformly mixing the elastomer and the cross-linker in a weight ratio of 50 : 1.

After the curing process at room temperature for 6 h, the prepared silicone rubber has a strong tendency to gain electrons and possesses excellent flexibility.^33^ The carbon nanotubes (CNTs) were dispersed in alcohol and stirred with a magnetic stirrer until the alcohol evaporated. Then, the CNTs were fully stirred in the silicone rubber mixture to prepare the conductive silicone rubber mixture. After the 24 h curing process at room temperature, the conductive silicone rubber was obtained. The CNTs mixed in the conductive silicone rubber help to form conductive pathways between the silicone rubber.^30^ The surface of the heat-shrinkable tube was sprayed with release agent that helps to separate the heat-shrinkable tube and silicone rubber.

### 2.2 Fabrication of CSTN

**Fig. 1:**
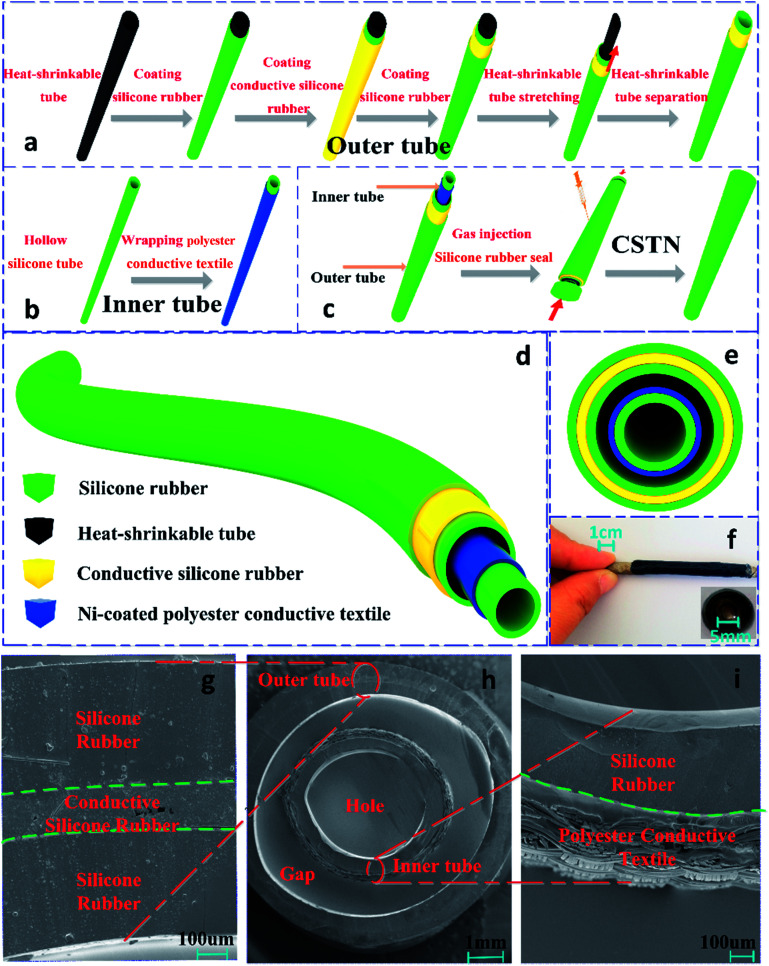
The structure schematic of core–shell coaxially structured triboelectric nanogenerator (CSTN). The fabrication processes for the outer tube (a), inner tube (b) and CSTN (c). (d) The structure schematic of CSTN. (e) The cross-sectional view of CSTN. (f) The digital photography of CSTN. (g–i) The cross-sectional SEM images of the outer tube, the CSTN and the inner tube.

The structure and fabrication processes for the core–shell coaxially structured triboelectric nanogenerator (CSTN) are illustrated in Fig. 1. The structure primarily includes two parts. One part is the inner-tube as the core (Fig. 1b), where the commercially produced hollow silicone tube is used as the flexible and stretchable substrate. The Ni-coated polyester conductive textile wrapped around the silicone tube works as both the triboelectric material and the inner electrode. The other part is the outer-tube as the shell (Fig. 1a), including the dielectric layer and its back electrode (outer electrode). The dielectric layer, as another triboelectric material and outer tube substrate, was prepared by coating the prepared silicone rubber mixture around the heat-shrinkable tube. Then, it was placed in a vacuum chamber for 20 min to remove bubbles and then cured at room temperature for 6 h.

Subsequently, the prepared conductive silicone rubber mixture was coated around the outer tube substrate and cured at room temperature for 24 h to form the outer electrode. The silicone rubber mixture was coated again to protect the CSTN from contamination. The heat-shrinkable tube was drawn and pulled away from the prepared outer tube, so that a hollow outer tube with a larger diameter (shell) is prepared. Following this, the inner tube (core) was inserted into the outer tube (shell). Air was injected into the space between the inner tube and the outer tube to form a gasbag. To maintain the air in the gasbag, the entire structure was encapsulated by the silicone rubber mixture (Fig. 1c). According to the above operation procedures, the triboelectric nanogenerator with a core–shell coaxial structure was prepared successfully (Fig. 1d–f). Fig. 1g–i show the cross-sectional scanning electron microscopy (SEM) images of the CSTN. A double-layer inner tube was nested in a three-layer outer tube; both the inner tube and outer tube are hollow structures with an air gap between the inner tube and the outer tube.

### 2.3 Electrical measurement of CSTN

The structure section of the CSTN was analysed by SEM. To measure the output performance of the CSTN, a linear motor was utilized to simulate the press-release process. The short-circuit current and open-circuit voltage of the CSTN were measured using a Keithley 6514 (pressing motion) and Keithley 2611B (bending motion and twisting motion). A pressure sensor (QLMH-P) and high-speed response display (QL-8016) were used to detect and display the pressure information.

## 3. Results and discussion

### 3.1 Working principle of CSTN

**Fig. 2:**
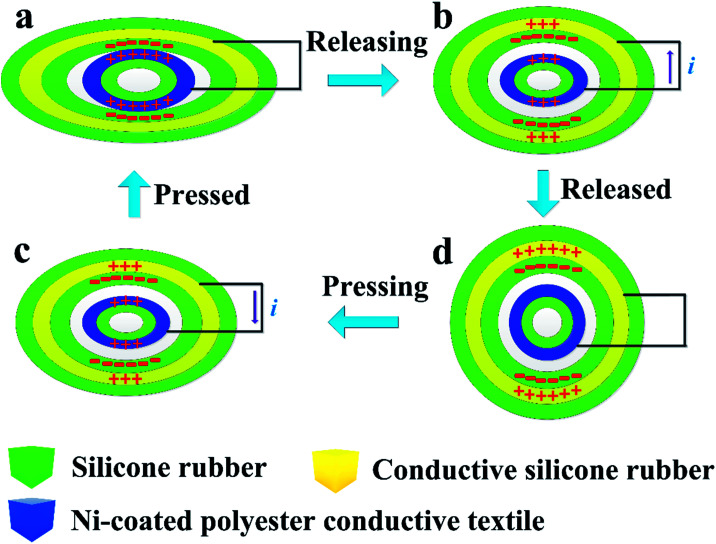
Schematic illustration of the power generation mechanism of CSTN. Triboelectric charge distribution of one current generation cycle: (a) full-contact state, (b) releasing state, (c) full-separation state (initial state) and (d) pressing state.

The power-generation mechanism of the core–shell coaxially structured triboelectric nanogenerator (CSTN) is illustrated in Fig. 2, which is based on the couple of triboelectrification and electrostatic induction,^35^ and the theoretical sources are derived from the Maxwell displacement current.^36^ The silicone rubber and Ni-coated polyester conductive textile are adopted as the negative and positive triboelectric materials due to their different electron affinity potential energy. In the initial state, without any deformation, no charge is transferred. The silicone rubber will contact with the inner electrode when the deformation occurs. Then, the silicone rubber will be charged negatively and the inner electrode will be charged positively owing to the different friction electrode sequence^29^ (Fig. 2a). As long as the deformation begins to recover, the separation between the silicone surface and the inner electrode will create an electric potential difference. The electrons will flow from the outer electrode to the inner electrode through the external circuit to balance the generated triboelectric electric potential (Fig. 2b). When the deformation is fully recovered, the electrons will flow back to the static equilibrium state (Fig. 2c). Subsequently, the electrons will flow from the inner electrode to the outer electrode when the deformation occurs again (Fig. 2d). Then, an alternating current will be generated in the external circuit through the reciprocating contact-separation motion.

### 3.2 Output performance of CSTN

**Fig. 3:**
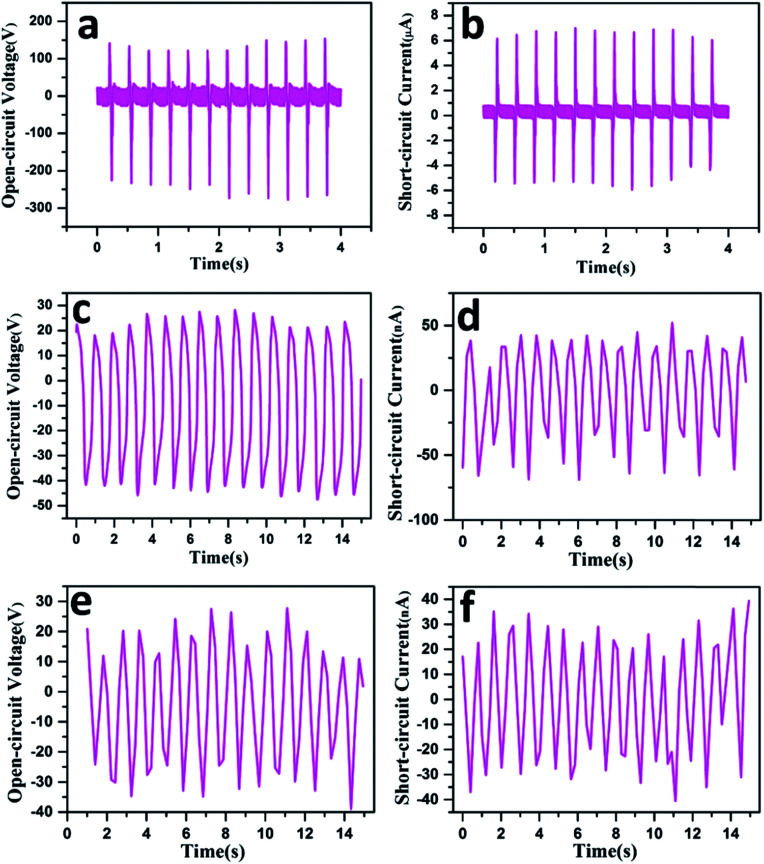
Output performance of the CSTN. (a) Open-circuit voltage and (b) short-circuit current of the CSTN under a given frequency of 3 Hz and pressure of 300 N. (c) Open-circuit voltage and (d) short-circuit current of the CSTN when bent at a 180-degree angle. (e) Open-circuit voltage and (f) short-circuit current of the CSTN when twisted at 360-degree angle.

To systematically investigate the output performances of the CSTN, a linear motor is utilized to simulate the press-release process. When the length of the CSTN is set at 6 cm and the inner tube diameter is set at 5 mm, an open-circuit voltage of 380 V and a short-circuit current of 11 μA can be obtained under a given frequency of 3 Hz and pressure of 300 N (Fig. 3a and b).

Due to the flexibility of the material and the axial symmetry of the structure, the designed CSTN can not only harvest energy from pressing motion, but also collect other types of energy, such as bending motion and twisting motion. When the CSTN is bent and twisted, the maximum open-circuit voltage can reach 65 V (Fig. 3c) and 50 V (Fig. 3e), and the maximum short-circuit current can reach 95 nA (Fig. 3d) and 60 nA (Fig. 3f), respectively.

**Fig. 4:**
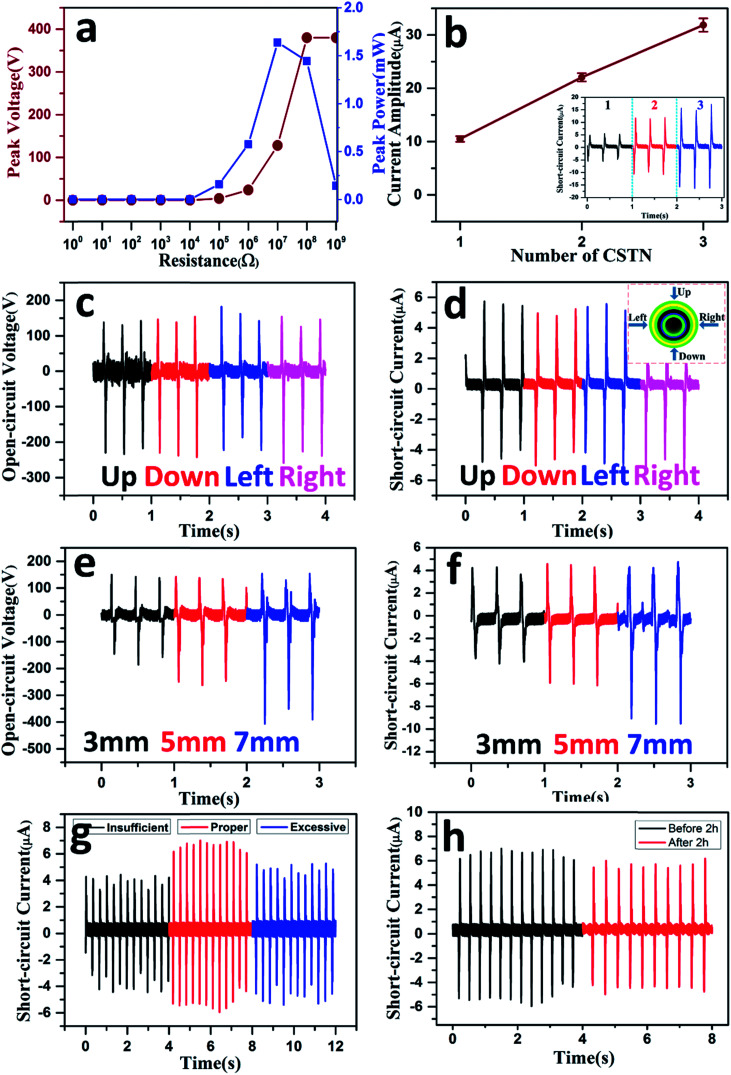
Output characteristics of the CSTN. (a) Dependence of the peak voltage and the peak power of a CSTN on the external load resistance. (b) Dependence of the current amplitude on the number of CSTN, the lower right inset shows the short-circuit current of the CSTN with a different number of tubes connected in parallel. (c) Open-circuit voltage and (d) short-circuit current of the CSTN under four pressure directions, the upper right inset shows the four different pressure directions perpendicular to the axis. (e) Open-circuit voltage and (f) short-circuit current of the CSTN with different inner diameters. (g) Short-circuit current of the CSTN with different gas saturation. (h) Short-circuit current of the CSTN before and after 2 h pressure process under a given frequency of 3 Hz and pressure of 300 N.

Fig. 4a displays the peak voltage and the peak power as a function of external load resistances. The peak voltage increases with an increase in resistance, while the peak power first increases and then decreases, reaching a maximum value of 1.638 mW at a load resistance of about 10 MΩ.

The short-circuit current can be linearly improved by means of multiple CSTN tubes connected in parallel (Fig. 4b). As the number of the CSTN increases from 1, 2 to 3, the peak short-circuit current increases from 11 μA, 21 μA to 31 μA (the inset in Fig. 4b). According to the characteristic of current multiplication, the CSTN can be used as an effective power source for the different power requirements of the electronic equipment, which can maximize energy utilization effectively.

Fig. 4c and d show that the output performance remains almost the same with vertical pressure from the four different directions (the inset in Fig. 4d). This is because of the unique symmetrical structure of CSTN. No matter which direction the pressure is applied, the contact area between the inner tube and the outer tube remains almost the same. The results demonstrate that the CSTN can be used as an energy generation unit to harvest energy as well as a sensing unit to acquire motion information from different directions and adapt for the diverse motion situation.

The contact area between the inner tube and the outer tube is a key factor that affects the output performance of the CSTN. When the length of the CSTN is set at 6 cm and the inner tube diameter increases from 3 mm, 5 mm to 7 mm, the open-circuit voltage increases from 300 V, 380 V to 480 V, respectively, and the short-circuit current increases from 8 μA, 11 μA to 13 μA (Fig. 4e and f). The reason for this can be explained by the larger diameter corresponding to a larger lateral area, which is equivalent to increasing the friction contact area between the Ni-coated polyester conductive textile and the silicone rubber. The CSTN with an inner tube diameter of 5 mm and length of 6 cm is adopted unless otherwise specified.

It is important to note that the gas saturation affects the output performance of the CSTN greatly (Fig. 4g). On the condition that excess gas is injected, the inner electrode and the silicone rubber may not be able to form a close contact upon deformation. In contrast, when gas is injected insufficiently, parts of the inner electrode and the silicone rubber may contact each other, affecting the output performance. However, because of the unique structure design and the flexibility of the silicone materials, the CSTN can generate electricity effectively as long as a certain amount of air is present.

For the frequent use of portable electronic devices, it is critical for the CSTN to maintain durability and stability for a long time in practical applications. Fig. 4h shows the output performance of the CSTN before and after a pressure process of 2 h under a given frequency of 3 Hz and pressure of 300 N, where the output performance is degraded only slightly. The results indicate that the CSTN has excellent stability and can be used in practical applications.

### 3.3 Applications of CSTN

#### 3.3.1 Power applications of CSTN

**Fig. 5:**
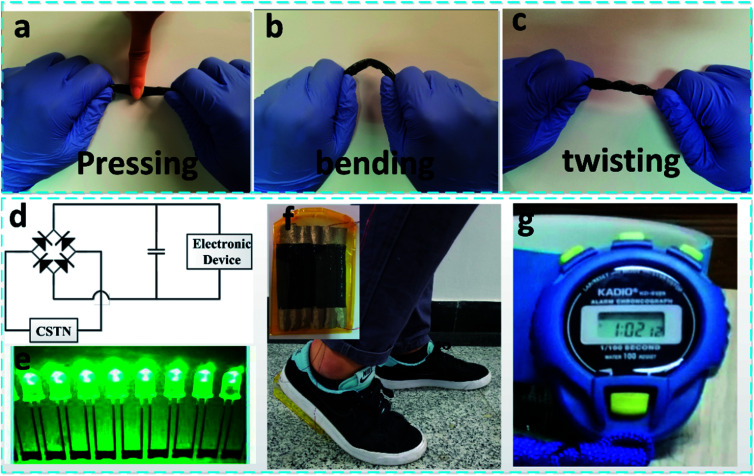
CSTN as an energy source. Schematic diagram demonstrating that the CSTN can collect various forms of energy: pressing motion (a), bending motion (b) and twisting motion (c). (d) Schematic diagram illustrating the drive circuit for electronics. (f) Photographic image of the CSTN fixed under the foot. The upper left inset shows the image of the six CSTNs connected in parallel. Illuminating devices (e) and portable electronics applications (g) of the CSTN.

In the daily life of human beings, motion energy can be collected from pressing motion (Fig. 5a), bending motion (Fig. 5b), twisting motion (Fig. 5c), and so on. The as-fabricated CSTN has broad applications for self-powered illuminating devices and portable electronics. When six CSTNs are connected in parallel and placed under shoes (Fig. 5f) along with a full wave bridge rectifier (Fig. 5d), the electric energy output through normal walking can light up 60 LEDs connected serially (Fig. 5e) and power up a competition timer (Fig. 5g), (ESI Videos 1 and 2†).

#### 3.3.2 Sensor applications of CSTN

**Fig. 6:**
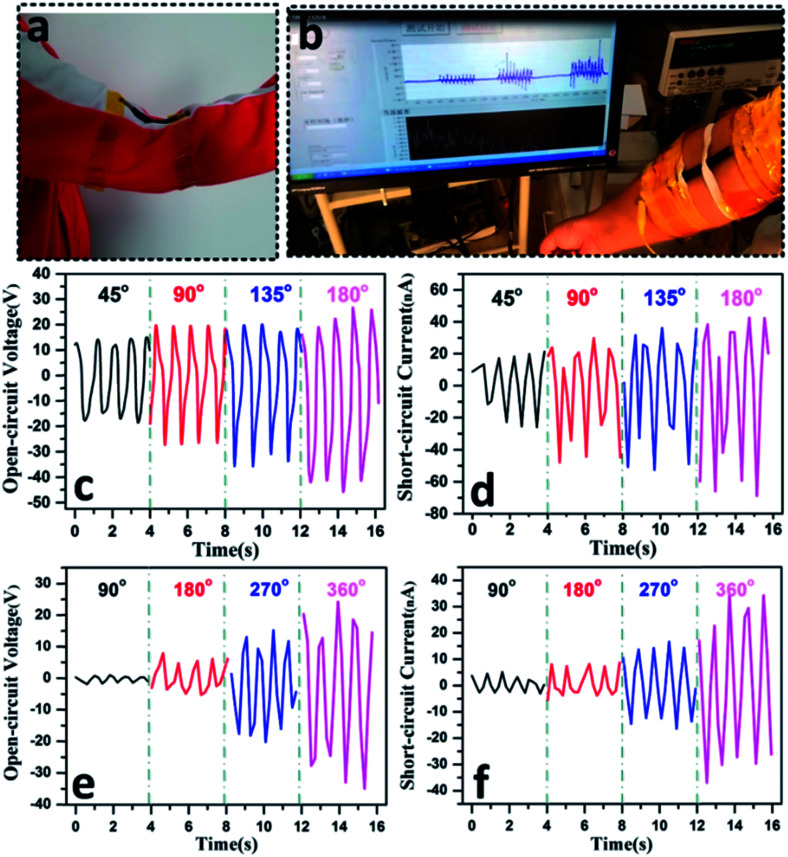
CSTN as a self-powered angle sensor for body motion sensing. (a and b) Self-powered angle sensor application of the CSTN for body motion sensing. (c) Open-circuit voltage and (d) short-circuit current of the CSTN under different bending angles. (e) Open-circuit voltage and (f) short-circuit current of the CSTN under different twisting angles.

When the CSTN is fixed at the elbow joint (Fig. 6a) and the arm is bent ten times at certain angles, as shown in the ESI Video 3,† the output performance gradually increases with increasing elbow joint bending angles (Fig. 6b). This observation can be explained by the fact that the Ni-coated polyester conductive textile and the silicone rubber will have closer contact and larger contact areas with the increase in the bending angles.

Fig. 6c–f present the signal outputs of a CSTN under various deformation angles. In the bending and twisting process, the energy generation mechanism of the CSTN is similar to that of a fixed-point press. When the bending angle is set from 45, 90, 135 to 180 degrees, the open-circuit voltage increases from 30 V, 45 V, 55 V to 65 V (Fig. 6c), and the short-circuit current increases from 45 nA, 60 nA, 75 nA to 95 nA (Fig. 6d), respectively. When the twisting angle is set from 90, 180, 270 to 360 degrees, the open-circuit voltage increases from 3 V, 10 V, 30 V to 50 V (Fig. 6e), and the short-circuit current increases from 8 nA, 11 nA, 27 nA to 60 nA (Fig. 6f), respectively.

As the CSTN could generate different electric signal outputs with different deformation angles, a simple bending or twisting angle sensor function can also be realized according to its signal outputs, which can be used for body motion sensing or health monitor.

## 4. Conclusions

In summary, a core–shell coaxially structured triboelectric nanogenerator (CSTN) was fabricated with the advantages of flexibility, light weight, sustainability and biological compatibility. The entire structure consists of an inner hollow circular tube as the core and outer hollow circular tubes as the shell, and is encapsulated by silicone rubber to protect the device from ambient contamination. A high short-circuit current of 11 μA and open-circuit voltage of 380 V can be obtained from a CSTN with a length of 6 cm, corresponding to a high peak power of 1.638 mW at a load resistance of about 10 MΩ. When six CSTNs are connected in parallel and placed under shoes, the electric energy output by normal walking can light up 60 LEDs connected serially and power up a competition timer. The CSTN can also generate different electric signal outputs under different deformation angles. Therefore, a simple angle sensor function can be realized, which indicates promising and broad application prospects in self-powered illuminating devices, portable electronics, motion sensing, health monitoring and so on.

## Author contributions

Zhumei Tian and Jian He contributed equally to this work.

## Conflicts of interest

There are no conflicts of interest to declare.

## Supplementary Material

RA-008-C7RA12739A-s001

RA-008-C7RA12739A-s002

RA-008-C7RA12739A-s003

RA-008-C7RA12739A-s004
