# Supplementary material for: Core–shell coaxially structured triboelectric nanogenerator for energy harvesting and motion sensing
Source: RSC Adv. 2018 Jan 15;8(6):2950–7. doi: 10.1039/c7ra12739a (PMC9077585; doi:10.1039/c7ra12739a)
Supplement: RA-008-C7RA12739A-s001 [file RA-008-C7RA12739A-s001.pdf]

Electronic Supplementary Material (ESI) for RSC Advances.
This journal is © The Royal Society of Chemistry 2017

Journal Name

ARTICLE

Supplementary Information

Core-Shell Coaxially Structured Triboelectric Nanogenerator for Energy Harvesting and Motion Sensing

Zhumei Tian [a,b], Jian He[a], Xi Chen[a], Tao Wen[a], Cong Zhai[a], Zengxing Zhang[c], Jundong Cho[a,d], Xiujian Chou[a], Chenyang Xue[a,*]

a Science and Technology on Electronic Test and Measurement Laboratory, North University of China, Taiyuan, 030051, China.

b Department of electronics, Xinzhou teachers university, Xinzhou,034000, China.

c Department of Micro and Nano Systems Technology, University College of Southeast Norway, Horten, 3184, Borre, Norway.

d Department of Electrical and Electronic Engineering, Sungkyunkwan University, Suwon, 16419,South Korea

*Corresponding author,

Prof. Chenyang Xue,

Address: NO. 3, College Road, Taiyuan City, Shanxi Province, China

Email: xuechenyang@nuc.edu.cn

This journal is © The Royal Society of Chemistry 20xx

Please do not adjust margins

Supporting Videos

1、Supporting video of lighting up 60 commercial LEDs when walking (fixed under the foot).

2、Supporting video of driving a competition timer when walking (fixed under the foot).

3、Supporting video of the motion sensing when elbow is bend in different angles.

This journal is © The Royal Society of Chemistry 20xx

Please do not adjust margins
